# Suppression of Somatosensory Evoked Cortical Responses by Noxious Stimuli

**DOI:** 10.1007/s10548-019-00721-z

**Published:** 2019-06-19

**Authors:** Nobuyuki Takeuchi, Tomoaki Kinukawa, Shunsuke Sugiyama, Koji Inui, Kousuke Kanemoto, Makoto Nishihara

**Affiliations:** 1 Neuropsychiatric Department, Aichi Medical University, Nagakute, 480-1195 Japan; 2 Department of Anesthesiology, Nagoya University, Nagoya, 466-8550 Japan; 3 Department of Psychiatry and Psychotherapy, Gifu University, Gifu, 501-1193 Japan; 4 Aichi Human Service Center, Institute of Human Developmental Research, Kasugai, 480-0392 Japan; 5 Department of Integrative Physiology, National Institute for Physiological Sciences, Okazak, 444-8585 Japan; 6 Multidisciplinary Pain Center, Aichi Medical University, Nagakute, 480-1195 Japan

**Keywords:** Change-related response, MEG, Sensory gating, Sensory suppression, Aδ, SII

## Abstract

Paired-pulse suppression refers to attenuation of neural activity in response to a second stimulus and has a pivotal role in inhibition of redundant sensory inputs. Previous studies have suggested that cortical responses to a somatosensory stimulus are modulated not only by a preceding same stimulus, but also by stimulus from a different submodality. Using magnetoencephalography, we examined somatosensory suppression induced by three different conditioning stimuli. The test stimulus was a train of electrical pulses to the dorsum of the left hand at 100 Hz lasting 1500 ms. For the pulse train, the intensity of the stimulus was abruptly increased at 1200 ms. Cortical responses to the abrupt intensity change were recorded and used as the test response. Conditioning stimuli were presented at 600 ms as pure tones, either innocuous or noxious electrical stimulation to the right foot. Four stimulus conditions were used: (1) Test alone, (2) Test + auditory stimulus, (3) Test + somatosensory stimulus, and (4) Test + nociceptive stimulus. Our results showed that the amplitude of the test response was significantly smaller for conditions (3) and (4) in the secondary somatosensory cortex contralateral (cSII) and ipsilateral (iSII) to the stimulated side as compared to the response to condition (1), whereas the amplitude of the response in the primary somatosensory cortex did not differ among the conditions. The auditory stimulus did not have effects on somatosensory change-related response. These findings show that somatosensory suppression was induced by not only a conditioning stimulus of the same somatosensory submodality and the same cutaneous site to the test stimulus, but also by that of a different submodality in a remote area.

## Introduction

A preceding sensory stimulus makes the response to a following stimulus small, a phenomenon sometimes referred to as suppression and widely studied (Jääskeläinen et al. 2011). By manipulating the conditioning-test interval (CTI), several temporally distinct inhibitory mechanisms have been shown (Inui et al. 2016), one of which is long-latency suppression induced with a CTI of 500–700 ms. This type of sensory suppression is usually observed in the auditory system by using paired-pulse suppression paradigms, in which two identical stimuli are successively presented, and is considered to represent processes that suppress redundant information. Such suppression is clinically important, because previous studies have shown deficits in paired-pulse suppression in patients with various conditions, including schizophrenia (Bramon et al. 2004; Patterson et al. 2008; Potter et al. 2006; Turetsky et al. 2007), bipolar disorder (Cheng et al. 2016), epilepsy (Becker et al. 2011), and attention-deficit/hyperactive disorder (Holstein et al. 2013).

As for the somatosensory system, similar to auditory paired-pulse suppression, responses to innocuous stimuli in the secondary somatosensory cortex contralateral to the stimulated side (cSII) are suppressed by insertion of a preceding stimulus prior to the test stimulus in healthy individuals (Arnfred et al. 2001; Nakagawa et al. 2014; Wühle et al. 2010). Several studies of clinical patients using somatosensory suppression paradigms have been conducted, including patients with schizophrenia, who were found to have deficits in SII suppression but not in the primary somatosensory cortex (SI) (Thoma et al. 2007). Although the degree of suppression showed a correlation between the auditory and somatosensory systems in healthy subjects (Takeuchi et al. 2018), patients with fibromyalgia were reported to have discrepancies between somatosensory and auditory suppression (Montoya et al. 2006).

It is well known that pain as well as cortical responses elicited by noxious stimuli are suppressed by innocuous somatosensory inputs (Inui et al. 2006b; Testani et al. 2015; Hayamizu et al. 2016). Although the underlying mechanisms are not fully understood, somatosensory stimulation such as peripheral nerve stimulation is an important tool for relief of pain in patients with chronic neuropathic pain (Johnson et al. 2015). Less is known about the effects of noxious input on cortical responses to an innocuous somatosensory input, though some studies have shown a significant impact of noxious stimulation on cortical responses to innocuous stimuli (Inui et al. 2006b) and tactile perception (Apkarian et al. 1994; Bolanowski et al. 2000), suggesting that somatosensory suppression is induced not only by inputs from the same modality but also a different submodality.

In addition to the hetero-submodal interaction, somatosensory perception and somatosensory evoked cortical responses are also known to be influenced by a conditioning somatosensory stimulus at sites different from that of the test stimulus (Greenwood and Goff 1987; Bolanowski et al. 2000; Hamada et al. 2001). In their study, Greenwood and Goff investigated somatosensory suppression with different peripheral nerves and showed interactions, while that of Hamada et al. found that responses in SII to stimulation of the left index finger were suppressed by a preceding stimulation to the right index finger. When test and conditioning stimuli are applied to the same cutaneous site, peripheral factors such as presynaptic events must be considered (Hashimoto and Kano 1998). However, when a conditioning stimulus of a different submodality or in a remote area is effective to modulate the test response, mechanisms other than simple recovery may be involved.

Change-related cortical responses are specifically elicited by abrupt changes in a continuous sensory stimulus, and can be clearly recorded u sing magnetoencephalography (MEG) or electroencephalography (EEG) without attention needed by the subject (Inui et al. 2010a, b; Nishihara et al. 2011, 2014; Yamashiro et al. 2011). These activities show high test–retest reliability (Inui et al. 2013; Otsuru et al. 2012; Kodaira et al. 2013), thus such measurement is considered to be reliable for examining higher order brain functions. Change-related activities are present in the somatosensory, visual, and auditory systems (Otsuru et al. 2011; Urakawa et al. 2010a, b; Tanaka et al. 2009a, b). Furthermore, a change-related cortical response is triggered by any kind of sensory change. For example, in the auditory system, very similar responses are elicited by changes in sound pressure, frequency, and location (Inui et al. 2010a, b; Yamashiro et al. 2011; Akiyama et al. 2011). Also, a test response elicited by an auditory feature change can be suppressed by a preceding stimulus caused by the change of another auditory feature (Inui et al. 2012). Therefore, any auditory feature change appears to activate a similar, or even identical, group of neurons relating to generation of the change-related response (Inui et al. 2012). In the somatosensory system, such a change-related response is elicited by stimulus onset and offset, as well as abrupt changes in stimulus intensity (Yamashiro et al. 2009; Otsuru et al. 2011), and considered to be triggered by changes on the body surface (Yamashiro et al. 2009).

Given that innocuous and noxious somatosensory stimuli activate very similar cortical regions (Mouraux et al. 2011) with very similar timing (Inui et al. 2003; Tanaka et al. 2008), and that activation in the somatosensory cortex mainly represents response to a new event (Tanaka et al. 2008; Mouraux et al. 2009; Otsuru et al. 2011), it is possible that somatosensory suppression is induced by any kind of preceding event on the body surface. To test that speculation, we examined long-latency somatosensory suppression using somatosensory change-related response with MEG. We recently developed a method to observe sensory suppression based on change-related cortical responses (Kodaira et al. 2013; Inui et al. 2012; Takeuchi et al. 2018). A change-related cortical response is specifically evoked by a sensory change, thus it is considered suitable to investigate the effects of other new sensory events. In the present study, we focused on two points, interactions between the innocuous and nociceptive somatosensory systems, and effects of the location of the conditioning stimulus on the body surface. Accordingly, we compared the effects of three types of conditioning stimuli, i.e., auditory, nociceptive, and innocuous somatosensory stimuli on cortical response to an innocuous somatosensory stimulus. In addition, we employed the right foot for the site of the conditioning stimulus, while the test stimulus was applied to the left hand.

## Methods

The study protocol was designed in accordance with the Declaration of Helsinki (World Medical Association 2008) and approved in advance by the Ethics Committee of the National Institute for Physiological Sciences, Okazaki, Japan. All subjects provided written consent prior to participation. Thirteen healthy volunteers (4 women, 9 men; age 23-40 years, mean 34.9 years) participated in this study. None had a history of mental or neurological disorders, nor substance abuse in the most recent 5 years, and all were free of medication at the time of testing.

## Stimulation

### Test Stimulus

For the innocuous test stimulus, a train of current-constant square wave pulses (pulse duration, 0.5 ms) at 100 Hz was applied to the dorsum of the left hand between the first and second metacarpal bones using a felt-tip bipolar electrode for transcutaneous electrical stimulation (TS). The total duration of the train stimulus was 1500 ms and the interval between trials was 2000 ms. The intensity of the stimulus was 1.2 times the sensory threshold until 1190 ms and then 1.8 times thereafter, with the result being that the abrupt increase in stimulus intensity at 1200 ms evoked change-related cortical responses.

### Conditioning Stimuli

**Fig. 1:**
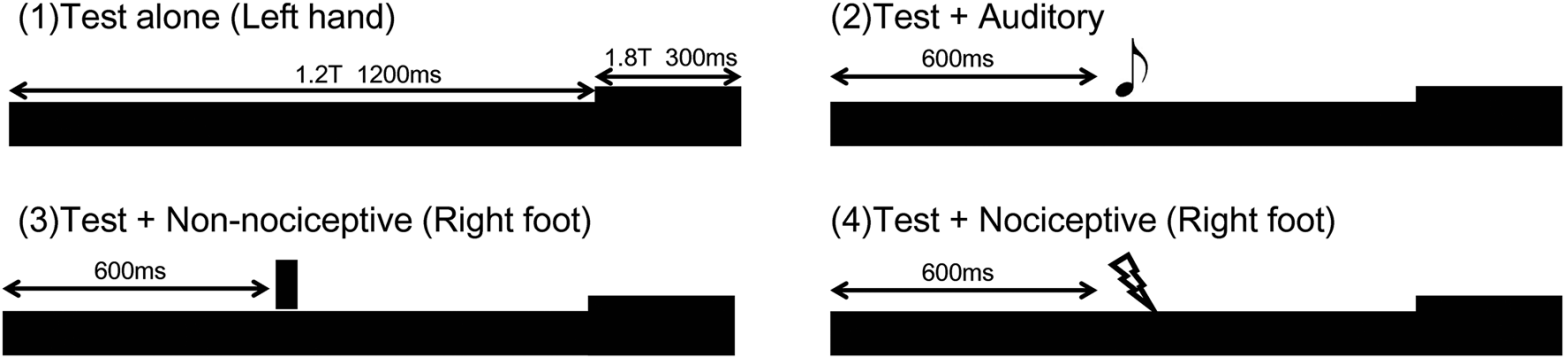
Paired stimulation paradigm using somatosensory change-related cortical responses. Stimulation paradigm for each stimulus. Conditioning stimuli were presented at 600 ms. T, sensory threshold

As a conditioning auditory stimulus, two 25-ms pure tones of 800 Hz (rise/fall, 5 ms) were presented at 600 ms without a space. The auditory stimulus was created by a personal computer (Panasonic CF-RZ6, Windows XP 32 bit) equipped with a sound card (SE-200PC, Onkyo, Osaka, Japan) and presented binaurally at a sound pressure level of 80 dB using earpieces (E-A-Rtone 3A, Aero Company, Indianapolis, IN). The intensity of the sound was measured with a 2-cc coupler (Electa, Tokyo) using a sound-level meter (EL-42, Rion, Tokyo) placed at the end of the tube. As a conditioning TS, we used an electrical stimulator (SEN-7203) and isolator (SS-J104, Nihon Kohden, Tokyo) to apply double current-constant square wave pulses (pulse duration, 0.5 ms) at 100 Hz to the dorsum of the right foot at 600 ms after the Test stimulus. The stimulus intensity was two times greater than the sensory threshold. Thus, the foot TS was of the same somatosensory submodality as that of the test stimulus, but applied to a different area and the opposite side. For a conditioning noxious stimulus, an intra-epidermal electrical stimulation (IES) method was used for selective stimulation of cutaneous A-delta fibers (Inui et al. 2002). That stimulus was triple pulses at 50 Hz with a 0.9-ms duration (rise/fall, 0.2 ms) and the stimulus intensity was 1.5 times greater than the sensory threshold (0.15 ± 0.06 mA) in each subject, and applied to the dorsum of the right foot at 600 ms after the onset of the Test stimulus. Thus, foot IES differed from the Test stimulus in regard to somatosensory submodality, spinal level, and side. Figure 1 shows the stimulation paradigm and evoked cortical responses in a representative subject.

## Procedures

We utilized four stimulus conditions; (1) Test TS alone, (2) Test TS + auditory stimulus, (3) Test TS + foot TS, and (4) Test TS + foot IES, which were randomly presented.

## Recordings

Each subject sat in a chair and watched a silent movie on a screen placed 2 m in front of them, and was instructed to ignore all stimuli throughout the experiment. Magnetic signals were recorded using a 306-channel whole-head type MEG system (Vector-view, ELEKTA Neuromag, Helsinki, Finland), which was comprised of 102 identical triple sensor elements. Each sensor element consisted of 2 orthogonal planar gradiometers and 1 magnetometer coupled to a multi-superconducting quantum interference device (SQUID), and thus provided 3 independent measurements of the magnetic fields. In the present study, we analyzed MEG signals recorded from 204 planar-type gradiometers, which were sufficiently powerful to detect the largest signal just over local cerebral sources. Signals were recorded with a bandpass filter of 0.1–330 Hz and digitized at 1000 Hz. Analyses were conducted from 100 ms before to 1700 ms after the onset of the stimulus. Epochs with MEG signals larger than 2.7 pT/cm in any gradiometer were automatically rejected from averaging. The four stimulus conditions were presented randomly, and at least 100 artifact-free epochs were averaged for each condition after rejection. The waveform was digitally filtered with a bandpass filter of 1.0–100 Hz.

## Analysis

The abrupt increase in intensity of the test stimulus at 1200 ms elicited clear magnetic responses in three sensor areas; the parietal area contralateral to the stimulation and temporal areas in both hemispheres. Initially, we calculated vector sums from the longitudinal and latitudinal derivatives of the response recorded on the planar-type gradiometers at each of the 102 sensor locations, which were obtained by squaring the MEG signals for each of two planar-type gradiometers at a sensor location, summing those squared signals, and then calculating the root of the sum (RSS) (Kida et al. 2007). This calculation was performed for each of the 102 sensor locations. Next, we used the obtained RSS waveforms and isocontour map of the RSS amplitude to search for a peak channel with the greatest amplitude for each prominent response, as the waveforms had a variety of responses with a different spatial distribution of amplitude. From those results, the peak amplitude and latency of the prominent responses in the RSS waveforms were measured at the peak channel. After obtaining four RSS waveforms for each location in each subject, one-way repeated measures ANOVA of the four conditions was performed.

Dipole analyses were performed using the Brain Electrical Source Analysis (BESA) software package (GmbH, Grafefling, Germany), as previously reported (Inui et al. 2004, 2006a). As described following, dipoles were estimated to be located in SI and SII. The obtained two- and three-dipole models were applied to MEG signals for all conditions to simplify the data analysis, then the peak latency and peak-to-peak amplitude for each cortical activity were measured using the source strength waveform. The first peak was defined as the greatest response between 30–80, 85–130, and 85–145 ms for SI, cSII, and iSII, respectively, while the second was defined as the polarity-reversed greatest response following the first peak. Percent suppression of the test response by the conditioning stimulus (% suppression) was calculated as follows: (Test alone response—(Conditioning + Test response)/Test alone response) × 100 (Takeuchi et al. 2017). Amplitude was compared among the four conditions using one-way ANOVA. When there was a significant difference, the amplitude of the response for the Test + conditioning stimulus was compared with that for the Test alone response using a paired *t* test with Bonferroni correction.

## Results

**Fig. 2:**
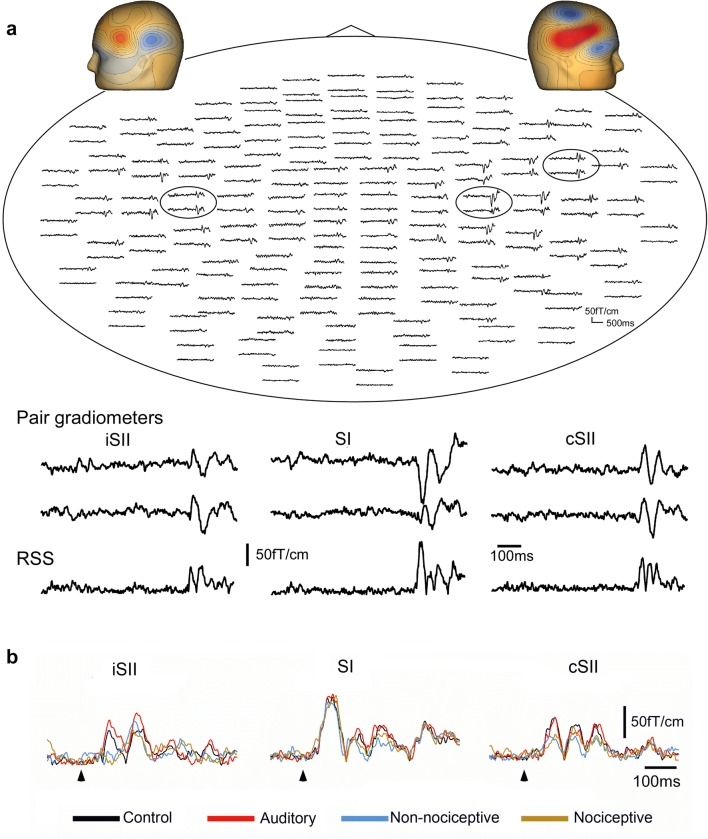
Sensor level analyses. **a** Top view trace of all sensors, enlarged waveform of selected sensors, and root sum square (RSS) waveforms obtained from selected sensor gradiometer, following the test stimulus in a representative subject. **b** Comparison of RSS waveforms among four events. Triangles indicate onset of test stimulus

**Fig. 3:**
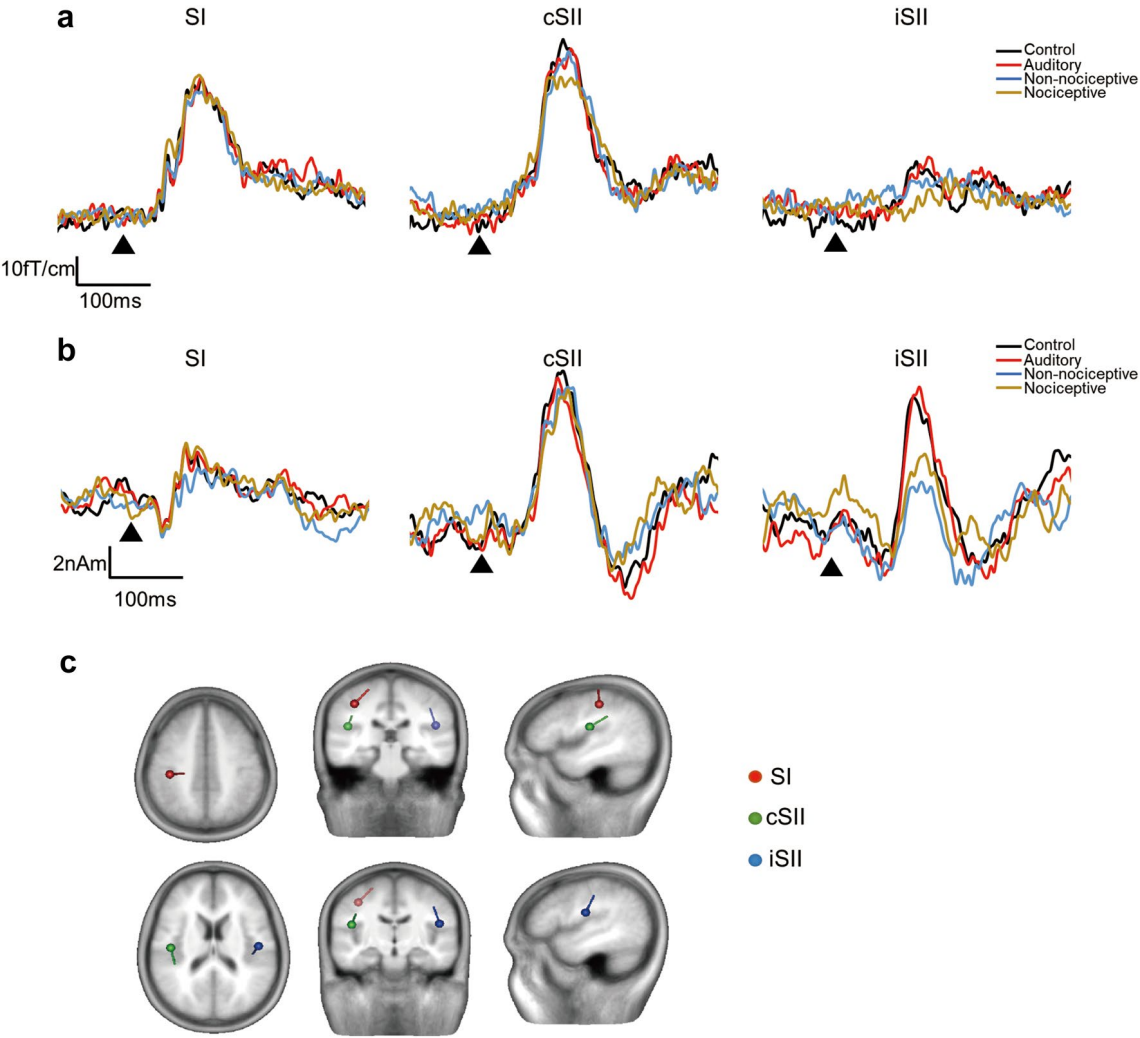
Grand-averaged source strength waveforms and dipole location. Grand averaged RSS waveforms (**a**) and source strength waveforms (**b**) for SI, cSII, and iSII are shown. Triangles indicate onset of test stimulus. **c** Mean locations of dipoles in SI, cSII, and iSII superimposed on slices of standard brain. Upper three slices show those for the SI dipole and lower slices for the iSII dipole

Magnetic responses to the test stimulus in a representative subject are shown in Fig. 2a. As demonstrated in the top view waveforms with a topography, there were three areas showing peaks of activity in both temporal areas and the right parietal area corresponding to the secondary somatosensory (SII) and primary somatosensory (SI) cortices, respectively. The procedures used for RSS analysis are shown in Fig. 2, including the selection of the sensor locations in SI, cSII, and iSII (2A), and waveforms of paired gradiometers and RSS waveforms (2B). Figure 3a shows grand-averaged RSS waveforms. Table 1 presents sensory threshold, peak amplitude, and % suppression values, while Table 2 shows latency in each brain area. The amplitudes of the SI (F_3,36_ = 0.39, p = 0.76), cSII (F_3,36_ = 0.42, p = 0.74), and iSII (F_3,36_ = 1.23, p = 0.31) activities did not differ significantly among the four conditions. Furthermore, the first and second peak latencies were not significantly different among the four conditions for SI (F_3,36_ = 0.59, p = 0.62; F_3,36_ = 0.62, p = 0.61, respectively), cSII (F_3,36_ = 0.69, p = 0.56; F_3,36_ = 0.26, p = 0.85), and iSII (F_3,36_ = 0.100, p = 0.41; F_3,36_ = 0.79, p = 0.51).

**Table 1:** Mean peak amplitude, percent suppression, and sensory threshold in each brain area in sensor level analysis. In this and following tables, data are shown in the mean (SD)

	Threshold (mA)	Amplitude (fT/cm)			% Suppression		
		SI	cSII	iSII	SI	cSII	iSII
Control	3.5 (1.5)	39.9 (23.4)	48.7 (37.2)	25.2 (11.9)			
Auditory		38.8 (23.6)	48.4 (38.1)	23.5 (11.9)	3.3 (19.0)	− 0.1 (24.6)	− 4.2 (50.0)
Non-nociceptive	8.9 (3.4)	37.0 (18.4)	46.3 (35.7)	25.1 (12.3)	0.3 (19.0)	0.0 (27.9)	− 5.2 (36.1)
Nociceptive	0.08 (0.04)	39.4 (21.3)	45.4 (35.6)	20.7 (10.2)	− 2.2 (24.0)	4.9 (25.3)	15.0 (29.8)

**Table 2:** Mean first and second peak latencies in each brain area in sensor level analysis

	Latency (ms)					
	SI		cSII		iSII	
	First peak	Second peak	First peak	Second peak	First peak	Second peak
Control	64.1 (14.8)	138 (20.6)	105 (12.6)	163 (28.4)	112 (13.8)	155 (16.1)
Auditory	67.4 (12.3)	134 (22.7)	108 (16.1)	168 (19.1)	113 (14.5)	158 (24.3)
Non-nociceptive	66.5 (12.6)	138 (24.7)	108 (12.2)	168 (19.1)	124 (31.9)	163 (33.9)
Nociceptive	64.2 (15.1)	135 (29.7)	104 (12.2)	164 (28.2)	117 (22.0)	167 (29.9)

**Fig. 4:**
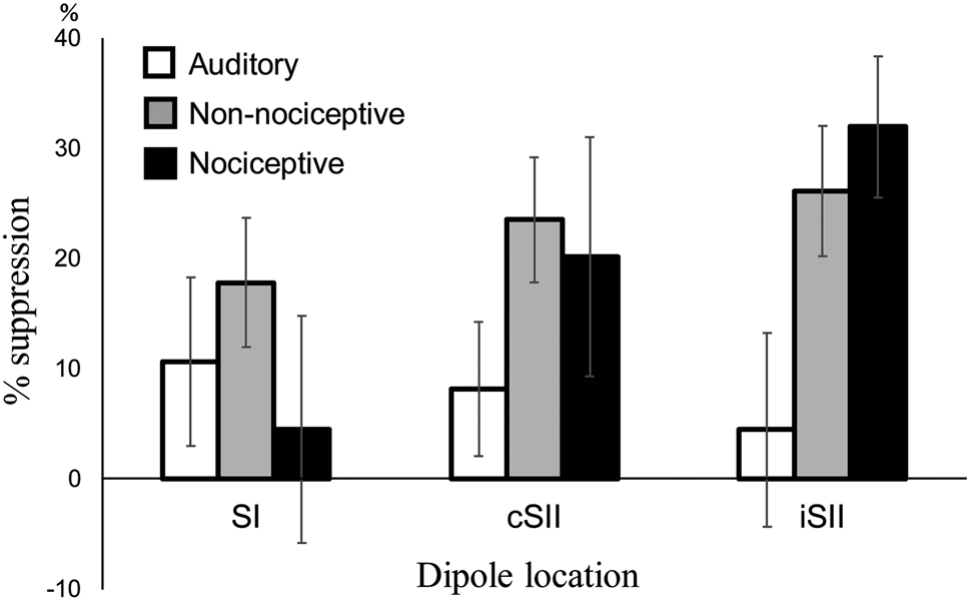
Mean percent suppression for each cortical activity. White, grey, and black bars represent suppression rates following auditory stimulus, TS to the foot (Non-nociceptive), and nociceptive IES to the foot (nociceptive), respectively. Vertical bars indicate standard error

Based on source analysis results, the dipoles were estimated to be located in the postcentral gyrus of the contralateral hemisphere (SI) and parasylvian region, including SII of both hemispheres. Among the 13 subjects, activities in SI, cSII, and iSII were detected in 11, 12, and 8, respectively, thus the analyses performed thereafter were based on the source strength waveforms obtained from these data. The grand-averaged source strength waveforms and mean locations of for each dipole are shown in Fig. 3b and c, respectively. The percent suppression for each condition is shown in Fig. 4. Values for the peak amplitude and latency are shown in Tables 3 and 4, respectively. The amplitude of the SI activity was not significantly different among the four conditions (F_3,30_ = 1.19, p = 0.33). On the other hand, the amplitude values for cSII (F_3,33_ = 6.42, p = 0.002, partial η^2^ = 0.37) and iSII (F_3,21_ = 4.22, p = 0.018, partial η^2^ = 0.38) were significantly different among the conditions. Furthermore, post hoc test findings showed that foot TS significantly attenuated the SII response for both cSII (p = 0.045) and iSII (p = 0.036), and foot IES also significantly attenuated the cSII (p = 0.005) and iSII (p = 0.027) responses. In contrast, the conditioning auditory stimulus did not have effects on the cSII (p = 0.36) or iSII (p > 0.9) amplitudes, and the first and second peak latencies did not differ significantly among the four conditions for SI (F_3,30_ = 0.168, p = 0.92, F_3,30_ = 1.00, p = 0.41, respectively), cSII (F_3,33_ = 1.84, p = 0.16; F_3,33_ = 1.87, p = 0.15), and iSII (F_3,21_ = 0.454, p = 0.72; F_3,21_ = 2.11, p = 0.13). Also, when % suppression was compared among the three conditioning stimuli, there were no significant differences among SI (F_2,20_ = 1.00, p = 0.39), cSII (F_2,22_ = 2.97, p = 0.72), and iSII (F_2,14_ = 2.42, p = 0.13).

**Table 3:** Mean peak amplitude and percent suppression in each brain area in dipole analysis

	Amplitude (nAm)			% Suppression		
	SI	cSII	iSII	SI	cSII	iSII
Control	10.8 (4.2)	22.6 (11.1)	15.7 (7.3)			
Auditory	9.9 (5.7)	20.7 (10.7)	15.7 (10.1)	10.6 (25.3)	8.1 (20.4)	4.5 (29.0)
Non-nociceptive	9.3 (5.4)	16.1 (5.8)	10.8 (5.5)	17.8 (20.2)	23.5 (20.0)	26.1 (31.0)
Nociceptive	10.7 (5.8)	17.6 (8.3)	10.8 (5.7)	4.5 (29.0)	20.2 (20.5)	31.9 (18.1)

**Table 4:** Mean first and second peak latencies in each brain area in dipole analysis

	Latency (ms)					
	SI		cSII		iSII	
	First peak	Second peak	First peak	Second peak	First peak	Second peak
Control	51.4 (18.6)	96.0 (59.5)	100 (21.0)	176 (27.7)	108 (17.8)	162 (28.8)
Auditory	52.3 (22.2)	92.6 (51.0)	105 (21.0)	168 (30.1)	104 (6.2)	160 (27.5)
Non-nociceptive	51.9 (19.5)	92.3 (51.4)	103 (22.4)	165 (21.6)	109 (17.6)	159 (26.1)
Nociceptive	52.7 (20.0)	94.1 (57.7)	107 (21.7)	163 (17.0)	110 (19.8)	153 (22.3)

The differing results between the dipole and sensor level analyses were considered to be mainly because of two factors. First, sensor level analysis was not able to separate activities from distinct origins and it is possible that overlapping activities from different groups of neurons made the effects of conditioning stimuli unclear. Second, for the sensor level analysis, we included data from all subjects, even when only weak activity detected. However, subjects with a low signal-to-noise ratio may have caused differences in the results between the dipole and sensor level analyses.

## Discussion

### Nature of Suppression

The present findings showed that a nociceptive stimulus induced long-latency somatosensory suppression. Few studies regarding short-latency somatosensory suppression of innocuous somatosensory responses by noxious inputs have been reported (Inui et al. 2006b) and this is the first such to show long-latency suppression. In contrast, a report with details of an opposite effect has been presented (Ploner et al. 2004). In that study, a conditioning pain stimulus induced by a laser facilitated responses in SII to an innocuous test stimulus that was applied to the same cutaneous area. Although there were some methodological differences between that and the present investigation, those for the test response might be important, as the previous study used an onset response, while change-related response was utilized in the present examinations. Given that change-related response is triggered by any new sensory event, our findings suggested that stimulus-driven activity is facilitated while change-driven activity is inhibited by a submodality different from the conditioning stimulus. This idea is consistent with other previous findings showing that noxious and innocuous somatosensory stimuli activated similar cortical regions (Tanaka et al. 2008; Mouraux et al. 2011; Omori et al. 2013; Frot et al. 2013) with similar timing (Inui et al. 2003; Tanaka et al. 2008). We consider that common change-related cortical activities among somatosensory submodalities account for a large portion of the cortical activations observed with noninvasive functional imaging techniques, such as MEG and fMRI. Therefore, it is thought rational to conclude that change-related responses have effects on following change-related responses.

This idea is supported by the present findings showing that both TS and IES applied to a cutaneous site remote from the test stimulation suppressed TS-induced change-relate responses. Because both of the conditioning stimuli were spatially different from the test stimulus in regard to spinal level and side, the results indicate that any somatosensory event at the body surface suppresses somatosensory change-related response. Although some studies have shown a somatotopic arrangement of SII neurons, it is less clear than that of SI (Maeda et al. 1999). In general, SII is considered to play a role in higher function, such as change detection, tactile leaning, and retention, rather than such sensory aspects as encoding the stimulus intensity or location (Hari and Forss 1999). For example, removal of SII in monkeys impaired tasks of tactile learning and retention, despite the finding that spatial alternation was not impaired (Garcha and Ettlinger 1978). In another study, the activation pattern of SII in humans showed a strong interaction between the right and left fingers (Hamada et al. 2001). Given the role of SII in change-detection (Otsuru et al. 2011), it seems likely that SII neurons respond to a new somatosensory event regardless of the site of stimulation, thus responding to an event prevents a full response to a following event. However, the degree of suppression may be different if the condition stimulus site changes and further studies are needed to elucidate effects related to its location.

A previous anatomical study showed connections between the somatosensory and auditory systems in Mongolian gerbils (Henschke et al. 2015), though little is known about their functional interaction. In humans, a few studies have been reported showing that cortical responses to tactile stimuli are influenced by auditory inputs (Sugiyama et al. 2018). Given the connection between the two systems, it is possible that the somatosensory cortex receives inhibitory modulation from the auditory cortex. However, to the best of our knowledge, no study showing suppression of somatosensory responses by sounds has been presented. Likewise, no significant effects of the sound were found in the present study, suggesting that projection from the auditory cortex to the somatosensory cortex is mainly facilitatory (Schroeder et al. 2001).

Other studies have reported attentional modulation of somatosensory processing (Bolton et al. 2011; Meftah et al. 2002), thus it is possible that attentional effects were involved in the present results. Nevertheless, all of the conditioning stimuli were presented at 600 ms prior to the test stimulus in the present study, thus the effects of attention were likely similar with every condition. Therefore, we considered that the absence of auditory effects showed a modest contribution of attention to long-latency suppression, at least under the present experimental conditions. This is important when such measures are used in clinical tests, because some patients have difficulty with maintaining attention. Although it was not significant, the sound reduced the Test alone response by 8%, which might be due to the effects of subject attention.

### Discrepancies Among SI and SII, and iSII

Conditioning stimuli attenuated SII activity but not that of SI in the present study, similar to results noted in previous studies of somatosensory long-latency paired-pulse suppression, which showed stronger effects on SII than SI (Wühle et al. 2010; Hamada et al. 2002). Additionally, findings of previous anatomical (Burton et al. 1995; Vogt et al. 1978) and electrophysiological (Inui et al. 2004; Pons et al. 1987, 1992) studies have demonstrated the presence of serial and hierarchical processing through SI and SII, suggesting greater or specific inhibitory mechanisms for SII. Some spatial overlapping in SII responses from different body parts has been found (Avanzini et al. 2018), thus SII is thought to be more sensitive to sensory changes than SI (Kodaira et al. 2013) and considered to play an important role in change detection.

Our results showed that suppression was stronger for iSII than cSII. The reason for this discrepancy may be related to the different components of cSII and iSII activities, because it is known that activities in the opercular region come from several distinct sources (Disbrow et al. 2000; Yamashiro et al. 2009). Particularly, differences between stimulus- and change-driven components appear to be important. An abrupt increase in stimulus strength was used to evoke change-related responses in the present study, thus SII responses should include both stimulus- and change-driven components, with the ratio of the two components possibly different between cSII and iSII. In previous studies, acute administration of nicotine enhanced change-related responses in iSII (Kodaira et al. 2013), while that had scant effects on stimulus-driven onset response (Otsuru et al. 2012). The neural origins are also different, as Yamashiro et al. showed that the dipole for stimulus-driven SII activity is located more anterior as compared to that for change-driven SII activity. As a candidate for the posterior source, they considered the temporo-parietal junction (TPJ), which is located more posterior than the stimulus-driven classical SII source and known to be sensitive to sensory changes (Downar et al. 2000, 2002). Although we did not separate stimulus-driven from change-driven components in the present study, this may explain our findings showing that suppression was greater for iSII, followed in order by cSII and SI.

In conclusion, the present study showed somatosensory suppression by conditioning stimuli from different somatosensory submodalities, spinal levels, and sides. In previous studies we used change-related cortical response to detect somatosensory suppression (Otsuru et al. 2011; Takeuchi et al. 2018). Because change-related response is dependent on the length of the preceding sensory event to be compared, haptic memory and comparison processes are involved in its generation (Otsuru et al. 2011), which also indicates that change-related response reflects a higher brain function. In addition, that response shows high test–retest reliability (Inui et al. 2013; Otsuru et al. 2012; Kodaira et al. 2013). We consider that the present paradigm is useful for objective observation of higher brain functions, including memory-based change detection and inhibitory interactions among somatosensory submodalities within a shorter inspection time period. Recently, we showed that the degree of suppression of change-related cortical response under a paired-pulse paradigm was correlated between the auditory and somatosensory systems, suggesting that those measures reflect inherent inhibitory abilities of individuals (Takeuchi et al. 2018). However, in some diseases, patients show deficits in a specific sensory system (Thoma et al. 2007; Montoya et al. 2006). Therefore, comparisons of suppression among several sensory modalities or among submodalities of a sensory system using a paired-pulse change-related response paradigm may be useful for clinical testing.

### Limitations

In the present study, the test stimulation was applied to the left side in all subjects, thus results showing that the inhibitory effect was greater for iSII than cSII might not have been due to effects based on the side of stimulation, but rather to functional differences between the left and right hemispheres. In addition, we were not able to clarify the effects of handedness because of the small sample size. Additional studies with a larger cohort are needed to elucidate these effects.
